# Automated Vehicle Counting from Pre-Recorded Video Using You Only Look Once (YOLO) Object Detection Model

**DOI:** 10.3390/jimaging9070131

**Published:** 2023-06-27

**Authors:** Mishuk Majumder, Chester Wilmot

**Affiliations:** Department of Civil & Environmental Engineering, Louisiana State University, Baton Rouge, LA 70803, USA; cecgw@lsu.edu

**Keywords:** automated vehicle counting, You Only Look Once (YOLO), object detection, TensorFlow, OpenCV, pre-recorded video, accuracy of automated traffic counting, benefit–cost (B/C) analysis

## Abstract

Different techniques are being applied for automated vehicle counting from video footage, which is a significant subject of interest to many researchers. In this context, the You Only Look Once (YOLO) object detection model, which has been developed recently, has emerged as a promising tool. In terms of accuracy and flexible interval counting, the adequacy of existing research on employing the model for vehicle counting from video footage is unlikely sufficient. The present study endeavors to develop computer algorithms for automated traffic counting from pre-recorded videos using the YOLO model with flexible interval counting. The study involves the development of algorithms aimed at detecting, tracking, and counting vehicles from pre-recorded videos. The YOLO model was applied in TensorFlow API with the assistance of OpenCV. The developed algorithms implement the YOLO model for counting vehicles in two-way directions in an efficient way. The accuracy of the automated counting was evaluated compared to the manual counts, and was found to be about 90 percent. The accuracy comparison also shows that the error of automated counting consistently occurs due to undercounting from unsuitable videos. In addition, a benefit–cost (B/C) analysis shows that implementing the automated counting method returns 1.76 times the investment.

## 1. Introduction

The act of automated vehicle counting can be executed through a variety of approaches, including applying in situ technologies and computer algorithms. In situ technologies refer to counting traffic using detectors located along the roadside [1]. There are different types of detectors in current use, such as pneumatic road tubes, piezoelectric sensors, magnetic loops, and microwave radar [2,3,4,5,6,7]. These technologies, however, come with significant expenses, and their accuracy depends on different factors such as weather (rain, fog, sun, and wind), the volume of traffic, and type of roads. A better alternative to these methods is using computer algorithms, which involve counting traffic automatically from pre-recorded video.

There are different traditional models for applying computer algorithms to count traffic. The basic procedure of all models is the application of the image processing technique [8]. The common methods for image processing are the traditional background subtraction method [9] and the sequential Monte Carlo method [10]. The background subtraction method involves separating the moving part of the image from the entire frame being analyzed [11]. The background subtraction method can be applied in different ways depending on the modeling of each pixel of the image. For example, Ridder et al. [12] (pp. 193–199) modeled each pixel of the background applying a Kalman filter to identify which pixels belong to the background and which do not, while Wren [13] (pp. 780–785) modeled the background using a single Gaussian value to estimate the probability that a pixel belongs to the background or not. However, these initial types of background subtraction methods have many limitations, such as not being robust when video footage contains shadows, low light, light changes, and slow-moving vehicles. In addition, the video processing speed of most of those methods is too slow for practical vehicle counting.

To overcome these problems, Stauffer and Grimson [9] (pp. 246–252) modeled each pixel as a mixture of Gaussian values, i.e., each pixel is modeled as a mixture of values. The method involves dividing each frame into several pixels and modeling each pixel based on certain rules. A single Gaussian is used for a pixel having a single lighting surface, whereas an adaptive Gaussian is used to approximate multiple surfaces and changing lighting conditions [14]. Objects are detected by grouping Gaussian values that do not match those of the previous frame using connected components. Moving objects are tracked from frame to frame to determine their direction of movement. However, the method is slow, performs poorly with large and overlapped objects, and only detects objects without classifying them.

Cucchiara et al. [15] (pp. 1–11) used a more robust method involving image processing and rule-based reasoning for vehicle tracking on visual data. This method is capable of tracking vehicles during the day or night. During the day, the spatio-temporal module extracts the blocks of pixels that move from frame to frame [16]. Their algorithm considers three consecutive frames to detect moving pixels. In this way, the vehicles are detected and tracked during the day. A high-level knowledge-based system is used to count vehicles, which separates the moving, stopped, and road-crossing vehicles for the desired vehicle counting purpose. At night, the system uses the morphological analysis of headlights to detect vehicles with headlight pairs. The algorithm separates headlight pairs from the image using image-masking and thresholding techniques. Headlight pairs are then matched with templates, and those that match are considered vehicles. However, this method cannot detect vehicles with a single headlight, such as motorcycles, and cannot classify vehicles.

Baoxin Li and Rama Chellappa [10] (pp. 530–544) applied a sequential Monte Carlo method for detecting, tracking, and verifying objects. This task is executed in three steps. Firstly, the current state of an object is defined by its position, density, and velocity. Secondly, sequential importance sampling (SIS) is used to identify objects in a particular frame [17]. Thirdly, the object is tracked from one frame to another frame and verified as a target object by setting the state ‘x’ to some parametrization (for example, the location of the object) of objects, which is determined in the first step. The results suggest that the algorithm provides a promising approach for tracking and verifying objects.

Removing unwanted shadows and classifying objects in an image was a great challenge for researchers in automated traffic counting. Hsieh et al. [18] (pp. 175–187) used a novel line-based shadow algorithm to solve these problems. In the first stage, vehicles are extracted from the background using image differencing, and these vehicles are passed through a shadow-elimination process to reduce shadows to a minimum level. A Kalman filter is used to track vehicles, which is accomplished based on the position and motion of the vehicles. The length and size of the vehicles are obtained through line fitting and connected component analysis. These two features are applied to categorize vehicles into different classes. This approach is an excellent method for tracking and classifying vehicles.

Lei Xie et al. [19] (pp. 883–886) propose a method for tracking objects from frame to frame. The method uses a scale-adaptive spatial appearance feature density technique, increasing object tracking sensitivity for both the scale and rotation scale. The image-tracking method can track objects in unfavorable imaging conditions by combining the object’s appearance with its background.

The advancement in deep learning has presented different object detection models, such as the Region-Based Convolutional Neural Network (R-CNN), Fast R-CNN, Single Shot Multi-Box Detector (SSD), and You Only Look Once (YOLO) [20]. R-CNN and Fast R-CNN, developed by Ross Girshick et al. [21] (pp. 580–587), use selective searches to detect objects. The Single Shot Detector (SSD), developed by Lui et al. [22] (pp. 21–37), is an object detection algorithm that utilizes a grid-based approach to divide an image into smaller regions, where each grid cell is assigned the task of detecting objects within its designated region. Redmon and Farhadi [23] (pp. 779–788) developed a different object detector called YOLO, which feeds the image once through the network and identifies the objects. In terms of speed and accuracy, the YOLO model performs better than other models [24,25]. YOLO uses the non-maximal suppression technique to process an image, which is an outstanding technique compared to other detectors. Initially, YOLO divides an input image into a 13 × 13 grid of cells and each cell predicts several bounding boxes in the image [23]. Then, YOLO algorithms estimate a single probability value for each bounding box. The acceptance of a bounding box as a valid object detection is controlled by a threshold value.

The performance of YOLO was found to be superior to that of other neural-network-based object detectors in terms of accuracy and speed [24,26]. The accuracy of the model reduces when the target video frame contains tiny objects. Since vehicle counts of this study do not involve any small object detections, the YOLO model performs well in this case.

Several studies have been conducted on in situ traffic counting technologies; for example, Patrizia Bellucci and Ernesto Cipriani [27] (pp. 175–187) applied a piezoelectric sensor; Patrick McGowen and Michael Sanderson [3] (p. 1) applied a pneumatic road tube; and Chen-Fu Liao [2] (p. 1) implemented inductive loops, which perform well in terms of accuracy of counting. These technologies are costly, so many traffic counting projects cannot afford them. This factor motivated this research to work on an economical automated vehicle counting method. Automated counting methods can be a good replacement for in situ technologies [28]. Many studies have been conducted on automated traffic detection and counting; for example, Baoxin Li and Rama Chellappa [10] (pp. 530–544) applied a sequential Monte Carlo method, Lei Xie et al. [19] (pp. 883–886) used a scale-adaptive spatial appearance feature density technique, Hsieh et al. [18] (pp. 175–187) applied a novel line-based shadow algorithm, Pereira et al. [29] (pp. 210–239) applied a LWR traffic flow model, Honghong Yang and Shiru Qu [30] (pp. 75–850) applied a background subtraction model with low-rank decomposition, Mattias Gustafsson and Sebastian Hjelm [31] (pp. 1–93) applied neural network models, Duc-Liem Dinh et al. [32] (pp. 1–15) applied edge computing methods, Amie Rosarie Cubeta Caballo and Chris Jordan Aliac [33] (pp. 150–154) applied a YOLO-based model for real-time tracking, and Subhrasankha Dey et al. [34] (pp. 1–14) applied a stochastic method. Though these studies conducted significant research on object detection and counting, some of these studies’ accuracy is not significant, some methods are not fast, and some methods cannot provide counts in flexible intervals. Compared to these studies, our proposed method is a significantly more efficient object detection model in terms of cost, speed, and accuracy that implements YOLO object detection model. Counting time intervals is also an important consideration, as many projects demand traffic data at varying intervals, such as 5 or 15 min intervals [35]. The proposed method can provide directional vehicle counts with flexible time interval counting, which is a major requirement for transportation planners. Therefore, the proposed method provides promising outcomes that will be valuable for the researchers, planners, engineers, and policymakers involved with transportation planning, design, and management, enabling them to obtain economical directional traffic counts with flexible time intervals. Moreover, the study will demonstrate a benefit–cost (B/C) analysis to investigate the benefit of the adaptation of the developed automated counting method.

## 2. Materials and Methods

### 2.1. Collection of Video Data

Ten strip mall business sites were selected to collect video data in the Baton Rouge metropolitan area, Louisiana, USA. The video footage of traffic movement was collected at the entrance and exit points of the strip malls using MioVision (Altanta, GA, USA); CountCam, and CountingCars (Spacksolutions, Minneapolis, MN, USA) cameras. The video was collected for two successive days from 8 AM to 6 PM for each site. Later, these video data were processed using the developed automated traffic counting method to evaluate the model’s performance.

### 2.2. Selection of YOLO Version

The authors selected YOLO version 3 for this research. This version is significantly faster, accurate, and user-friendly [24]. However, four new versions of YOLO models are available after the release of YOLO version 3, which are YOLOv4, YOLOv5, YOLOv6, and YOLOv7. Compared to versions 4, 5, and 6, YOLO version 7 is the most accurate [36,37]. The reason for the higher accuracy is the resolution capability of the model. YOLO version 7 processes an image with a resolution of 608 pixels by 60 pixels, whereas YOLO version 3 processes an image at a resolution of 416 by 416 [37,38]. This higher resolution enables version 7 to detect tiny objects in the image and provides higher accuracy than version 3. Since vehicle counting does not involve any tiny object detections, the results of the proposed method using YOLO version 3 do not affect the result.

Since the proposed method does not involve any tiny object detection, the research results are also not affected by the other compared methods. For example, a recent (2023) version of the stochastic method proposed by Subhrasankha Dey et al. [34] (pp. 1–14) shows that the model can reach accuracy up to 90%, where our proposed model provides consistent accuracy of 90 percent. In addition, a recent version of the background subtraction model proposed by Honghong Yang and Shiru Qu [30] (pp. 75–850) 2017 showed an accuracy of 89.4 percent. This model still provides less accuracy, and the speed is slower than that of YOLO version 3. Moreover, a vision-based vehicle counting method proposed by Keith A. Redmill [39] (pp. 5086) shows an accuracy of 88.8 percent. Though the other in situ technologies, such as piezoelectric sensors, pneumatic road tubes, and inductive loops, provide more accurate counts, these methods are costly. Therefore, the other compared methods do not affect the proposed method results.

Thus, in this study YOLOv3 [40] (pp. 1–6) was selected for the development of automated vehicle counting method. The YOLO model was applied with TensorFlow [41] (pp. 265–283) and OpenCV [42] (pp. 120–123) libraries for vehicle detection and counting. Python was used for developing all the algorithms.

### 2.3. Conversion of YOLO Weight File to TensorFlow API

The YOLO weight file was originally developed using the C/C++ programming language. In order to apply the model in the Python preferable environment, the weight file was converted to TensorFlow architecture. The required packages for this conversion were TensorFlow 18.0 (a deep learning library), Numpy (a package of routines in Python that support many mathematical functions), OpenCV (an open-source computer vision library), TQDM (a progress bar library that provides useful routines for nested loops), and Python. The open-source YOLO weight file was downloaded from the official YOLO website (pjreddie.com) [43]. After that, the directory of the downloaded YOLO weight file was provided under the root project directory ‘./data/darknet_weights/’, in the command prompt [43]. The ‘python convert_weight.py’ command was run from the project directory to convert the YOLO weight file to the TensorFlow format. The reason for using Python instead of compiling and running a Darknet trainer from the C source is the convenience of the project and working with the Python programming language.

### 2.4. Settings for Input Files

First, this step defines the directory of the YOLO weight file, anchor definitions, YOLO model class definition, and the number of graphics processing units available for use. After that, the arguments were developed for the name and directory of the input pre-recorded video footage using the ‘parser’ task. All these developed arguments were executed using the function ‘__main__’, the fundamental function that manages the vehicle detection algorithms.

The input video files must be processed before uploading to the program. All video files must be converted to the .mp4 format. If there are multiple video files, it is suggested that the video files be joined together because multiple video files cannot be uploaded simultaneously in the program. After processing the video file, it is uploaded in the section titled ‘input’ by dragging and dropping the file. The video file’s name is manually typed before the ‘.mp4’ title in the algorithm. When the program is executed, it checks for all the defined arguments and the input video file directory. If the program validates the directory and title of the video, it moves to the processing step.

### 2.5. Detection of Vehicles

**Figure 1:**
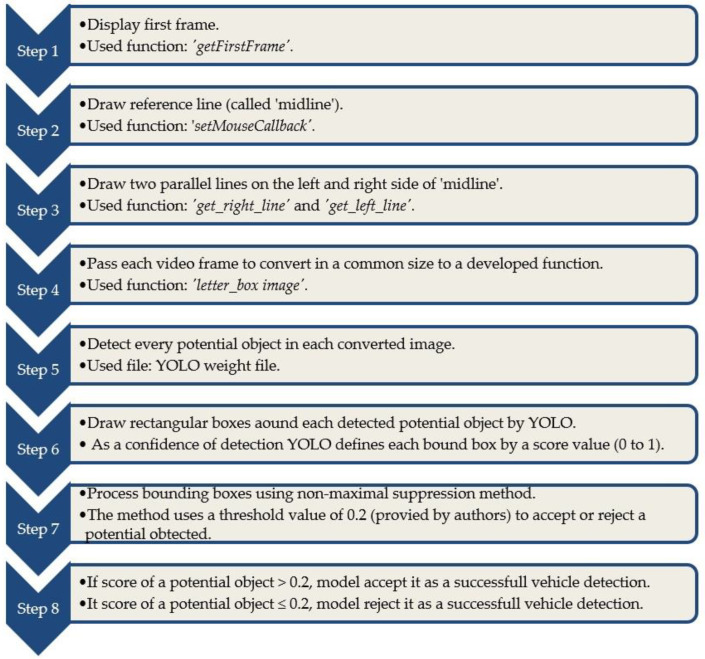
The flow chart diagram of a successful vehicle detection by YOLO.

In this section, algorithms were developed to draw reference lines and detect vehicles in each video frame. The algorithms allow users to draw a reference line with simple mouse clicks when the very first video frame appears. The reference lines validate the program to make a successful vehicle count in the later steps. Though the detection of vehicles in each video frame was conducted by applying the converted YOLO weight file, a few algorithms were developed to execute the detection process, for example, the conversion of each image to a common size and providing threshold a value to identify potential detected objects. The total vehicle detection process is shown in Figure 1.

**Figure 2:**
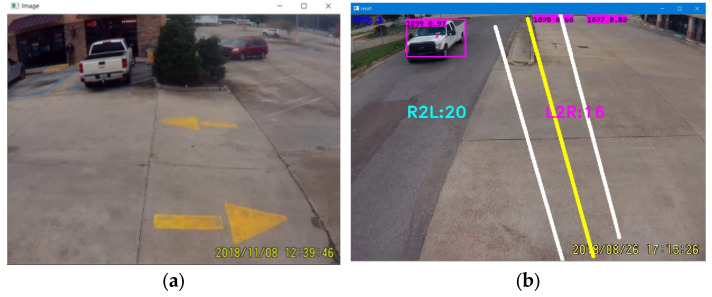
The visualization of the first frame, mid-line, and parallel lines by the program. (**a**) The first frame of a video file displayed by the program. (**b**) A mid-line as displayed in yellow color and two parallel lines in white color, drawn by the user.

When the input argument receives the name and the path of an input video file, the program calls the detector function (‘detect_start’) and passes the file information. Then the program calls another function (‘getFirstFrame’) to capture and display the first frame with the help of OpenCV, as shown in Figure 2 [44]. After that, the developed algorithm calls the mouse handling function (‘setMouseCallback’), which allows a user to select the starting and ending point of a reference line with simple mouse clicks. The algorithms automatically connect two points and draw a reference line, as shown in Figure 2. According to the developed algorithms, the reference line is titled ‘mid_line’. Afterward, the ‘while True’ (a developed conditional loop statement) is executed to open every video frame.

After that, the program calls the ‘detect_image’ function. The parameters of this function are a single video frame and reference lines. Under this function, a few more functions are defined, such as ‘get_right_line’ and ‘get_left_line’, which draw parallel lines on the right side and left side of the ‘mid_line’, respectively, as shown in Figure 2. Typical geometry-based arguments are applied to draw these parallel lines, and the program automatically draws parallel lines when a user draws the mid-reference line.

The ‘detect_image’ function passes each video frame to the function ‘letter_box image’. This function converts each video frame to a common size (128 × 128 pixels) as required by the converted YOLO weight file in the TensorFlow API. Then, TensorFlow calls the converted YOLO weight file to detect objects in each frame [45,46,47]. At this stage, the YOLO weight file detects every potential object in an image. Following a successful detection of an object in an image, YOLO draws a rectangular box surrounding the object. As a result, the outputs of a processed image are rectangular bounding boxes and the score of the corresponding boxes. A score value means a detected object’s confidence level, which lies between 0 to 1. The bounding boxes are processed by YOLO using the non-maximal suppression method [23,48]. The controlling factor in this process is a threshold value, which screens out the bounding boxes with a low confidence interval. This study used a threshold value of 0.2, meaning that bounding boxes with a score higher than 0.2 are accepted for further processing and vice versa. The sorted bounding boxes are sent to the ‘dets’ array to track detected objects.

### 2.6. Tracking Vehicles

Since video footage consists of thousands of frames (i.e., images), an object must be tracked from one frame to another to determine its direction of movement [18]. The main task of this section is to develop algorithms to track the detected object frame by frame. The sorted bounding boxes from the ‘dets’ array are tracked using the ‘KalmanBoxTracker’, which compares the current frame with the immediately previous frame using the pixel variance of the frame [19,49]. When it finds a similarity in pixel values, it updates the object (i.e., bounding box), memorizes it for consideration in the next frame, and repeats the process.

**Figure 3:**
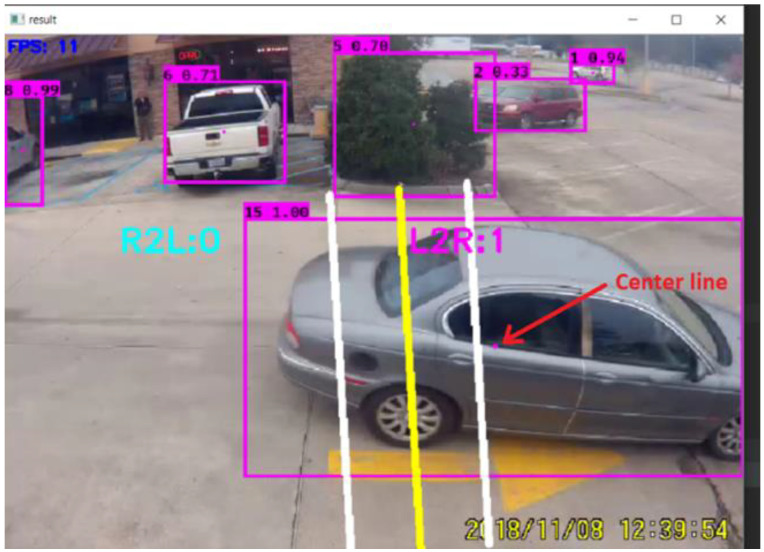
A typical dotted centerline as displayed as a red dot and shown by a red arrow, and rectangular bounding boxes as displayed in purple color.

In this research, the tracker entitles each bounding box using a numeric value such as 1, 2, and 3. Rectangles are drawn around each tracked object using the function ‘draw.rectangle’, which was developed in the study. The rectangles are displayed on the computer screen, as shown in Figure 3. The purpose of these rectangular boxes is to draw a dotted line in the center of the boxes that leads to counting vehicles, which will be discussed more in the counting vehicle section. A typical centerline is shown in Figure 3, as marked by the red arrow.

### 2.7. Settings for Output File

In this step, algorithms were developed to obtain the vehicle counts in a spreadsheet format with a flexible time interval and desired direction. This study requires counting vehicles in a flexible real-time interval, while YOLO’s counting speed differs from the real-time clock. To solve this problem, YOLO evaluates the number of frames in the desired time interval and the time to process a single frame. Then it estimates the total processing time of all frames in the desired time interval and considers that as the YOLO time interval. The algorithms are capable of providing vehicle counts in entry and exit directions.

### 2.8. Algorithm for Counting Vehicle

The algorithms determine the number and directions of counts based on the passing direction of the dotted center line through the mid-line and parallel line. For example, when the center line intersects the left parallel line first, the program confirms that the vehicle is coming from the left side. It can be assumed that the left side direction is the entry direction. Afterward, the program considers it a successful entry count when it intersects the mid-line. The developed ‘leftToright_counter’ and ‘rightToleft_counter’ functions count vehicles from the left and right sides, respectively.

**Figure 4:**
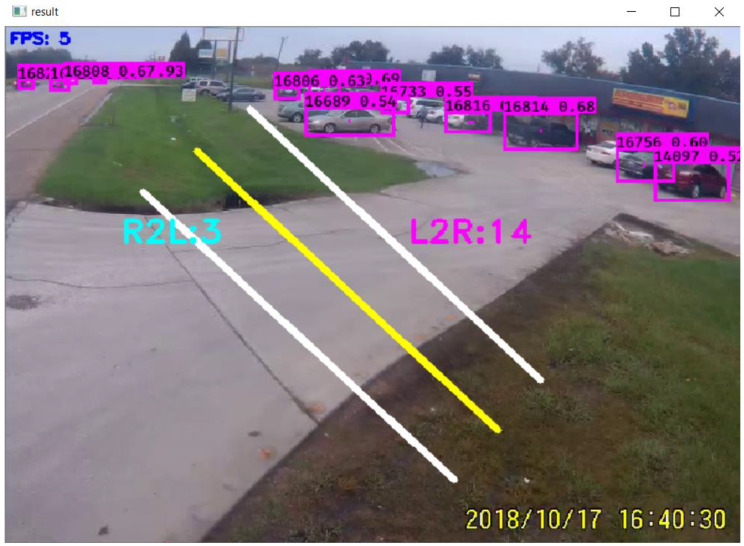
Vehicle counts displayed on screen as shown as R2L and L2R.

Counts are provided in a spreadsheet with the desired time interval. In addition, the counts are displayed on the computer screen. Figure 4 shows typical entry and exit counts for strip malls. This figure assumes that when vehicles are coming from right to left, they are departing the strip mall, and when approaching from left to right, they are entering the parking lot. Figure 4 shows the exit counts as R2L and the number of counts is three vehicles, and entry counts as L2R, where the number of counts is fourteen.

### 2.9. Apply YOLO with OpenCV and CUDA

In order to allow YOLO to operate in a GPU environment at increased video processing speed, the model must be applied with OpenCV (Open-Source Computer Vision Library) before running the program. To accomplish this task, two open-source programs, cuDNN (a deep neural network-based library that provides graphics processing unit functionality) and CUDA (NVIDIA’s programming language used to code the graphics card), are required. Both cuDNN and CUDA can be downloaded from the NVIDIA website (developer.nvidia.com, accessed on 5 June 2019) [50]. CUDA must be installed properly since the success of the application of YOLO with OpenCV and cuDNN depends on the correct installation of CUDA. The success of the installation can be checked by running a sample video file in the program. If the installation is successful, the graphics properties are shown at the bottom of the program as a response to the installation.

### 2.10. Embedded Hardware Platforms

The counting speed of the program depends on the configuration of the hardware platform. The following hardware platform was used in this study:

Processor: Intel(R) Core (TM)i7-8750CPU @ 2.20 GHz 2.21 GHz;

RAM: 2.7 GHz, 16.0 GB;

GPU: NVIDIA GeForce GTX 1060, 6 GB.

In this platform, it was found that the program takes about one hour forty minutes to one hour fifty minutes to process an hour of video. Using a high-configuration computer for processing videos is recommended to achieve increased video processing speed. A 2060 GPU with a video memory of more than 6 GB is recommended for timely processing.

### 2.11. Benefit–Cost Analysis

Lastly, a benefit–cost (B/C) analysis was conducted to evaluate the benefit of the automated counting method for practical implementation [51]. The video data collection and manual counting from the pre-recorded video were considered the minimum benefit of B/C analysis of automated counting [52]. Three types of cameras were used to collect video data: a MioVision Camera, a CountCam2 Traffic Recorder, and a CountCam. The manual counts were conducted from pre-recorded videos using a computer. In this research, it was found that it takes 21 min to manually count an hour of video by the optimum increase in video player speed. Using this ratio, the total video hours were converted to actual working time. The actual working hours were converted to monetary value using an hourly pay rate. Another important factor is the electricity consumption while conducting manual counts by trained individuals. This factor is also included in the estimation of minimum benefit. The per kilowatt electricity cost of the research area (East Baton Rouge Parish, LA, USA) is USD 0.13 [53]. This rate was used to convert the kilowatt hours taken for conducting manual counts to a monetary value. The cost of cameras and computers is long-term investment, and this is also the case for both manual and automated counting methods. So, this factor was disregarded for the benefit–cost (B/C) analysis. Therefore, the minimum benefit of the project includes the following factors:

1. Cost of field data collection;
2. Cost of conducting manual counts;
3. Cost of energy consumption by computers.

The video data collection method and cost for the automated counts are the same as for the manual counts. Automated counting is conducted by computer algorithms using a hardware platform, and an individual is appointed to upload videos to the computer program. The individual is paid for the hours taken to upload videos in the program. In addition, before uploading videos, the individual pre-processes video footage, such as converting all videos to .mp4 format and joining videos. After uploading videos to the program, the computer is left for hours to process videos. From this research experience, it was found that it takes 15 min to process and upload 10 h of video to the program. This factor was applied to convert video hours to working hours. After that, the hourly pay rate was used to estimate the cost of automated counting.

There is considerable energy consumption in working with deep learning models. According to Neil C. Thompson et al. [54] (pp. 50–55), the deep learning model involves enormous computational costs. This research has two computational costs: the energy consumption cost for configuring the computer and setting the model; and the energy consumption cost for automated counts. First, the hours and kilowatt hours taken for configuring the computer and setting the model, and automated counts, are estimated. After that, the unit price of electricity, i.e., 0.13 USD/kW-h [53], is used to convert the hours to the monetary value. The cost of the automated counting method includes the following factors:

1. Cost of field data collection;
2. Cost of conducting automated counts;
3. Energy cost of configuring computer and setting model;
4. Energy cost for running the model for video processing.

Equation (1) was used to estimate the benefit–cost (B/C) ratio of the automated counting method.

(1) $$B/C=\frac{Benefit}{Cost}=\frac{\geq (\Sigma_{K}^{k}H_{v}{+}_{K}^{k}H_{m}+{kWh}_{m})}{(\Sigma_{K}^{k}H_{v}{+}_{K}^{k}H_{a}+{kWh}_{con}+{kWh}_{a})}\approx \;\frac{\geq (\Sigma_{K}^{k}C_{v}{+}_{K}^{k}C_{m}+E_{m})}{(\Sigma_{K}^{k}C_{v}{+}_{K}^{k}C_{a}+E_{con}+E_{a})}$$

k denotes a site;

K denotes the total number of data collection sites;

$H_{v}$ = Working hours for collecting videos (h.);

$H_{m}$ = Hours for conducting manual counts (h.);

$H_{a}$ = Hours for conducting automated counts (h.);

${kWh}_{m}=$ Kilowatt hours for manual count (kWh);

${kWh}_{con}=$ Kilowatt hours for configuring computer and setting model (kWh);

${kWh}_{a}=$ Kilowatt hours for automated count (kWh);

$C_{v}$ = Cost for collecting videos (USD)

$C_{m}$ = Cost for conducting manual counts (USD);

$C_{a}$ = Operating cost for conducting automated counts by trained individuals (USD);

$E_{m}$ = Energy consumption cost for manual counts (USD);

$E_{con}$ = Energy consumption cost for configuring the computer and setting the model (USD);

$E_{a}$ = Energy consumption cost for automated counts (USD).

## 3. Results

### 3.1. Automated Counts

The pre-recorded videos were analyzed using the developed computer algorithms. Since the video was collected from 8 AM to 8 PM for each site for both day 1 and day 2, the total duration of video for an access point of a site was 20 h. The total number of access points for all 10 sites was 16. So, the total duration of video for all ten sites was 320 h. Therefore, in this automated counting method, a total of 320 h of video was processed. The entry and exit counts applying the automated counting method for the ten sites are shown in Table 1.

### 3.2. Accuracy Evaluation

Automated counts were compared with manual counts to estimate the accuracy of the automated counting. The accuracy of a daily entry, daily exit, and total counts of individual sites for day 1 and day 2 are shown in Table 2 and Table 3, respectively.

### 3.3. Paired t-Test

A two-tailed paired-*t*-test is a suitable statistical analysis to evaluate the differences between the observations of two variables. So, a two-tailed paired *t*-test was performed to evaluate the similarity between manual and automated counts. In this research, manual and automated counts were performed on ten sites. In this case, manual and automated counts are considered two variables, and sites are considered the same subject of interest.

A few assumptions were established for this test. It was assumed that independent variables (i.e., sites) consist of two related groups (i.e., manual and automated counts), there are no significant outliers in the differences between manual and automated counts, and the distribution of differences between manual and automated counts shows an approximately normal distribution. The following hypotheses were considered:

H_0_: N_ai_ = N_mi_∀i (2)

H_A_: N_ai_ ≠ N_mi_∀_I_ (3)

N_ai_ denotes automated total daily vehicle count at site *i*;

N_mi_ = manual total daily vehicle counts at site *i*.

The paired *t*-test was conducted for two cases for day 1 and day 2, individually. In the first case, the *t*-test was conducted for the difference between manual and automated counts (i.e., manual–automated). In the second case, the test was conducted for the adjusted difference between manual and automated counts, where the adjustment was performed by the means of the difference (i.e., manual—automated—mean). This second case was introduced to observe the effect of removing the undercounting bias in the results. The test results are shown in Table 4, Table 5, Table 6 and Table 7.

In the case of the difference between manual and automated counts, i.e., Table 4 and Table 6, the null hypothesis is rejected for both day 1 and day 2. So, it is found that manual and automated counts are significantly different. In the case of the adjusted difference between manual and automated counts, i.e., Table 5 and Table 7, the null hypothesis could not be rejected. So, it can be interpreted that manual and automated counts are not significantly different from each other if the consistent undercounting of the automated method is removed.

However, one of the assumptions of a two-tailed paired *t*-test is that data shows a normal distribution pattern. Since the sample size of the paired *t*-test is small, a normality test was conducted to see whether the sample shows a normal distribution. In this case, the differences between manual and automated counts for day 1 and day 2 were individually analyzed. From the test, it was found that the data for the difference between manual and automated counts for day 1 and day 2 do not show a normal distribution. The reason for not showing normal distribution is random sampling and the small data size. Therefore, it may be a potential reason for not showing the similarity of manual and automated counting data in the paired *t*-test.

### 3.4. Confidence Limits

Confidence intervals were estimated for the difference between paired manual and automated counts for day 1 and day 2, individually. The results are shown in Table 8 and Table 9 for day 1 and day 2, respectively. From Table 8, it can be observed that the upper confidence limit is 55.82 and the lower limit is 32.38. So, it can be interpreted that there is a 95 percent chance that the true mean difference between paired observations will be in the range 55.82, 32.38, and there is a 5 percent chance that the true mean will not be in the range 55.82, 32.38.

Table 9 shows that the upper confidence limit is 57.33 and the lower limit is 32.07. From this confidence limit, it can be implied that there is a 95 percent chance that the true mean difference between paired observations will be in the range 57.33, 32.07, and there is a 5 percent chance that the true mean will not be in the range 57.33, 32.07.

### 3.5. Benefit–Cost Analysis

The benefit–cost (B/C) analysis was conducted using Equation (1), as provided in the methodology section. The details quantity of the B/C analysis parameters are as follows.

Pay rate of trained individuals = 10 USD per hour;

Unit per of kWh energy (East Baton Rouge parish, LA, USA) = USD 0.13 [53];

The hardware device used in this research is Lenovo legion Y7000P-1060 and the energy consumption is 183.96 Wh. [55];

K denotes the total number of data collection sites = 10 sites;

$H_{v}$ = Working hours for collecting videos = 160 h;

$H_{m}$ = Hours for conducting manual counts = 112 h;

$H_{a}$ = Operating hours for conducting automated counts = 16 h;

${kWh}_{m}=$ Kilowatt hours for manual count (kWh)

  = energy consumption in 1 h * total hours

  = 183.96 Wh. * 112 h = 20.60 kWh;

${kWh}_{con}=$ Kilowatt hours for configuring computer and setting model (kWh)

  = energy consumption in 1 hr. * total hours

  = 183.96 Wh. * 48 h = 8.83 kWh;

${kWh}_{a}=$ Kilowatt hours for automated counts (kWh)

  = energy consumption in 1 h * total hours

  = 183.96 Wh. * 480 h = 88.30 kWh;

$C_{v}$ = Cost for collecting videos= 160 * USD 10 = USD 1600;

$C_{m}$ = Cost for conducting manual counts = 112 * 10 = USD 1120;

$C_{a}$ = Operating cost for conducting automated counts by trained individuals (USD)

 = 16 * USD 10 = USD 160;

$E_{m}$ = Energy consumption cost for manual counts (USD)

 = kWh * unit price = 20.60 * USD 0.13 = USD 2.68;

$E_{con}$ = Energy consumption for configuring computer and model setting

  = kWh * unit price = 8.83 * USD 0.13 = USD 1.15;

$E_{a}$ = Energy consumption cost for automated counts (USD)

  = kWh * unit price = 88.30 * USD 0.13 = USD 11.18.

Using Equation (1) and the factors described above, the benefit–cost (B/C) analysis is conducted as follows:

$$B/C=\frac{Benefit}{Cost}=\frac{\geq (160\;h+112\;h+20.60\;kWh}{\left(160\;h+16\;h+8.83\;kWh+88.30\;kWh\right)}\approx \frac{\geq (\$1600+\$1120+\$2.68)}{\left(\$1600+\$160+\$1.15+\$11.18\right)}=1.54$$

The estimated B/C ratio is 1.54, which implies that the proposed automated counting method has a 54 percent greater benefit then the traditional manual counts.

### 3.6. Comparison with Other Methods

The accuracy of different automated counting methods, including this study, is shown in Table 10.

Pneumatic road tube counting: Patrick McGowen and Michael Sanderson [3] (p. 1) conducted a study to evaluate the accuracy of the pneumatic road tube counter, where the authors found that the accuracy was about 99 percent [3]. In addition, the average error in a daily traffic count might be near zero; the absolute error of a typical 15 min count averaged closer to 10% [3]. These results suggest that the inaccuracy level is masked by the positive and negative counting errors canceling each other out. Errors in speed and classification were much more significant. These results raise questions about the reliability of pneumatic road tube counters in accurately measuring traffic volumes.

Piezoelectric sensor: When a vehicle passes over a piezoelectric sensor, mechanical energy passes to the sensor, thereby converting mechanical energy to electrical energy, which is analyzed for vehicle counting. The accuracy of traffic counts applying the piezoelectric sensor is reportedly 99 percent [27] (pp. 175–187).

Inductive loops: According to Chen-Fu Liao [2] (p. 1), the loop signature system could obtain more accurate, reliable, and comprehensive traffic performance measures for transportation agencies. The author conducted several tests on inductive loops to find the accuracy of traffic counts, and it was found that the accuracy was about 99 percent [2] (p. 1).

Computer algorithms: Mattias Gustafsson and Sebastian Hjelm [31] (pp. 1–93) developed algorithms to conduct automated traffic counts from pre-recorded videos. They collected high-resolution videos for automated traffic counts because it increases the success of counts. They trained and evaluated several neural network models that detect and count vehicles in various scenes and achieved accuracy above 90% [31].

Pereira et al. [29] (pp. 210–239) also developed a computer algorithm for automated traffic counting, emphasizing the speed and accuracy of counts. The authors reported that the accuracy of their program varies from 60 percent to 70 percent. The study reported that low-resolution video is responsible for the failure of the program to count traffic.

Developed method: The algorithms developed in this study can provide 90 percent accurate counts, which is significant compared to other computer algorithms. Although the accuracy of pneumatic road tube counting, piezoelectric sensors, and inductive loops is higher than that of the developed algorithms, these technologies are expensive, especially for small projects. Therefore, the developed algorithms can be a suitable alternative regarding cost and accuracy.

### 3.7. Limitations of Program

**Figure 5:**
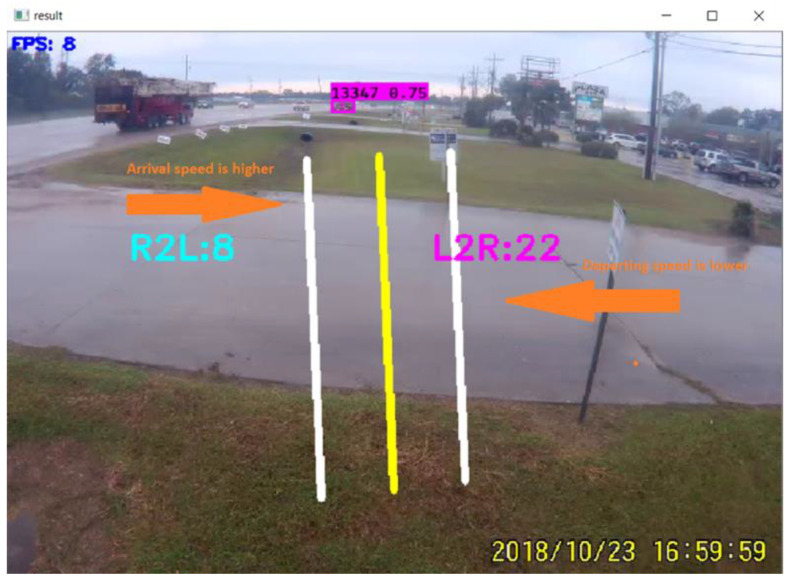
Error due to arrival and departure speed.

The program is not efficient enough when vehicles pass at high speed because vehicles appear for a short time in the footage, and the program does not have enough time to detect the vehicle. Figure 5 explains this scenario in the case of an entrance of a strip mall, where vehicles enter from the major road to the parking lot at high speed, and the program sometimes fails to detect vehicles. Conversely, the vehicles pass slowly and even stop in a queue when exiting the parking space. In this case, the program is very efficient in detecting and counting vehicles.

**Figure 6:**
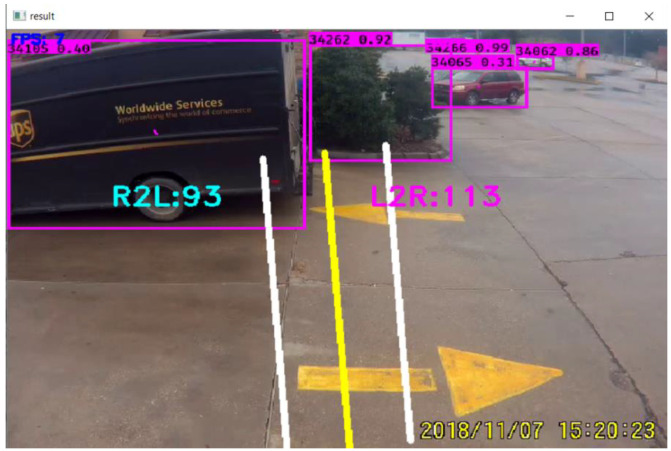
Unsuitable camera angle and frame.

The camera angle is a significant reason for reducing the accuracy of counts. When the camera is very close to the entrance, vehicles appear on a large scale and cover most of the frame; sometimes, some parts of the vehicle remain out of the frame. Figure 6 shows that the camera is very close to the entrance and does not cover the full view of the entrance. As a result, the UPS vehicle appears large, and most of the parts remain out of the frame. So, it is not the ideal frame for efficient counting.

Raindrops obscure the camera lenses, resulting in a bad-quality video recording, and the program cannot count accurately.

Visibility is an important factor that controls the quality of the video, and low visibility results in poor video quality and inefficient counting. The causes of low visibility are rain, low light, evening recording, and cloudy weather.

The program cannot detect overlapped vehicles, which results in undercounting. This happens when two vehicles arrive or depart simultaneously, and overlap in the video frame.

The counting speed of the program varies depending on the hardware platform. The counting speed increases with the configuration of the computer.

## 4. Discussion

The proposed automated vehicle counting model implements the YOLO object detection model with the assistance of OpenCV for vehicle counting from pre-recorded videos. The total error, classification error, and interval error of the proposed automated counting method were evaluated by comparing the automated counts with the manual counts and statistical analysis. The accuracy of automated counting was found to be about 90%, where it was observed that the errors occur due to undercounting of vehicles. The accuracy is consistent and significant compared to that of other methods. The automated analysis indicates that processing an hour of video takes about one hour and thirty minutes. The proposed model can be useful for real-time manner implementation for different applications such as traffic management systems, Intelligent Transportation Systems (ITSs), traffic congestion monitoring, parking management, traffic safety analysis, and transit planning. It was found from the study that the proposed model has some excellent advantages, such as providing traffic counts with 90 percent accuracy in a consistent manner; providing directional vehicle counts with flexible time intervals as required by many engineers, planners, and policymakers; and being economical, especially for small transportation projects. On the other hand, the proposed model has some drawbacks, such as the program not being efficient enough when vehicles pass at high speed, and the program compromises accuracy when the video quality is poor, especially when recorded during rain, low light, evening, and cloudy weather. In addition, the wrong camera angle and vehicles overlapping during recording may reduce the model’s accuracy. To overcome the drawbacks, it is recommended to use high-resolution cameras to obtain good-quality videos, properly install the cameras to avoid oscillation due to wind, and clean the camera lens before every installation. Moreover, it is recommended to use high-resolution computers to increase the speed of counting and save energy. A benefit–cost analysis was conducted considering different factors, including the energy consumption of the system. From the benefit–cost (B/C) analysis, the B/C was found to be greater than or equal to 1.54, which means the automated counting method has 54 percent greater benefit than the traditional manual counting method. In other words, it can be implied that the model can be suitable for the practical application of vehicle counting, especially for projects that cannot afford in situ counting technologies.

## 5. Conclusions

This study presents an efficient method of automated vehicle counting from pre-recorded video by applying a pre-trained YOLO object detector in TensorFlow API. The accuracy of automated counting was found to be about 90 percent, with automated counting consistently being undercounted. This accuracy is very significant compared to the other automated counting method. The average processing speed for an hour of video was found to be about one hour and thirty minutes. The advantage of the model is that it can provide 90 percent accuracy of directional counts with a flexible interval time in a consistent manner. On the other hand, the drawback of the model is that it compromises accuracy when the vehicles pass at high speed and overlap, the video frame is narrow, and the video quality is poor. From the benefit–cost (B/C) analysis, the B/C was found to be greater than or equal to 1.54, which implies that the automated method improves the benefit by 54% compared to the traditional manual method. Therefore, the developed algorithm might be an excellent replacement for the other expensive automated counting methods.

## Figures and Tables

**Table 1:** Automated vehicle counting results (vehicle per day).

Site No	Site Name	Day 1		Day 2	
		Entry	Exit	Entry	Exit
1	6031 Siegen Ln, 70809	295	269	268	235
3	3148 Ambassador Caffery Pkwy	481	485	571	564
5	1712 SW Railroad Ave, Hammond, LA 70403	172	168	164	162
11	28811 Walker South Rd, Walker, LA 70785	154	145	182	181
15	5635 Main St B, Zachary, LA 70791	335	331	281	264
18	1551 US-51 BUS, Ponchatoula, LA 70454	114	124	112	131
21	13711 Coursey Blvd	212	164	229	184
23	14210 Airline Hwy, 70737	162	145	121	112
28	13394 LA-73, Geismar, LA 70734	145	134	132	124
32	17134 Hwy 44, 70769	134	124	118	109

**Table 2:** Accuracy of automated vehicle counts for day 1.

Site	Entry			Exit			Total		
	Manual	Automated	Accuracy (%)	Manual	Automated	Accuracy (%)	Manual	Automated	Accuracy (%)
1	309	295	95.47	287	269	93.73	596	564	94.63
3	526	481	91.44	528	485	91.86	1054	966	91.65
5	196	172	87.76	194	168	86.60	390	340	87.18
11	172	154	89.53	164	145	88.41	336	299	88.99
15	354	335	94.63	346	331	95.66	700	666	95.14
18	149	114	76.51	153	124	81.05	302	238	78.81
21	224	212	94.64	181	164	90.61	405	376	92.84
23	180	162	90.00	165	145	87.88	345	307	88.99
28	162	145	89.51	144	134	93.06	306	279	91.18
32	159	134	84.28	141	124	87.94	300	258	86.00

**Table 3:** Accuracy of automated vehicle counting for day 2.

Site	Entry			Exit			Total		
	Manual	Automated	Accuracy (%)	Manual	Automated	Accuracy (%)	Manual	Automated	Accuracy (%)
1	288	268	93.06	259	235	90.73	547	503	91.96
3	617	571	92.54	610	564	92.46	1227	1135	92.50
5	194	164	84.54	186	162	87.10	380	326	85.79
11	207	182	87.92	200	181	90.50	407	363	89.19
15	297	281	94.61	284	264	92.96	581	545	93.80
18	142	112	78.87	145	131	90.34	287	243	84.67
21	247	229	92.71	209	184	88.04	456	413	90.57
23	132	121	91.67	124	112	90.32	256	233	91.02
28	158	132	83.54	149	124	83.22	307	256	83.39
32	129	118	91.47	114	109	95.61	243	227	93.42

**Table 4:** Paired *t*-test result for the difference between automated and manual counts for day 1.

Parameter	Value	
Mean	44.10	
Standard deviation	18.91	
Standard deviation of mean	5.98	
T stat	7.37	
95% C.I.	30.12	59.27

**Table 5:** Paired *t*-test for the difference between automated and manual counts adjusted by mean (manual-automated-mean) for day 1.

Parameter	Value	
Mean	−0.60	
Standard deviation	18.91	
Standard deviation of mean	5.98	
T stat	−0.10	
95% C.I.	−14.57	14.57

**Table 6:** Paired *t*-test result for the difference between automated and manual counts for day 2.

Parameter	Value	
Mean	44.70	
Standard deviation	20.38	
Standard deviation of mean	6.44	
T stat	6.93	
95% C.I.	30.12	59.27

**Table 7:** Paired *t*-test for the difference between automated and manual counts adjusted by mean (manual-automated-mean) for day 2.

Parameter	Value	
Mean	−2.84217 × 10^−15^	
Standard deviation	20.38	
Standard deviation of mean	6.44	
T stat	−4.41008 × 10^−16^	
95% C.I.	−14.57	14.57

**Table 8:** Confidence limits for the difference between paired observations for day 1.

Parameter	Value
Mean	44.10
Standard deviation	18.91
Sample size	10
Confidence coefficient (95%)	1.96
Margin of error	11.72
Upper confidence limit	55.82
Lower confidence limit	32.38

**Table 9:** Confidence Limits for the Difference Between Paired Observations for Day 2.

Parameter	Value
Mean	44.70
Standard deviation	20.38
Sample size	10
Confidence coefficient (95%)	1.96
Margin of error	12.63
Upper confidence limit	57.33
Lower confidence limit	32.07

**Table 10:** Accuracy of different automated counting methods.

Method Name	Accuracy	Comment
Pneumatic road tube counting	99 percent	Absolute error of a typical 15 min count averaged closer to 10%
Piezoelectric sensor	99 percent	
Inductive loops	90 percent	
Pereira et al. [29] (pp. 210–239)	60 to 70 percent	Computer algorithms for automated counting
Mattias Gustafsson and Sebastian Hjelm [31] (pp. 1–93)	90 percent	Computer algorithms for automated counting from pre-recorded video
This Study	90 percent	Computer algorithms for automated counting from pre-recorded video by applying YOLO

## Data Availability

The data of this study are available at https://github.com/mmajum2/Traffic-Counting-YOLO.git, accessed on 3 June 2023. Any other data is obtainable upon request from the corresponding authors for academic research.
